# Is digital health technology empowering patients?

**DOI:** 10.1002/jmrs.20

**Published:** 2013-08-22

**Authors:** Leslie Robinson

**Affiliations:** Directorate of Radiography, University of SalfordGreater Manchester, UK

Reflecting on personal observations and drawing on examples from the literature, this editorial will explore how digital health information is creating the “empowered” patient.

There is a saying that *knowledge is power* and searching the Internet I discover that this saying is attributed to Francis Bacon; thus, the Internet can provide knowledge and, if the saying holds true, has the potential to be an empowering entity. In much the same way, patients are searching for information on the Internet and are gaining information about their conditions that was previously only accessible to health professionals. Notwithstanding the fact the there is a huge range in the quality of available information, what is important is that patients *believe* they are more informed, whether their sources are valid or not, ergo they should feel more empowered to contribute in decisions regarding their care. Before considering the implications of such an argument, let us first understand *how* the Internet is changing the nature of patient information, first by considering the format of *static information* and second by the way in which Web 2.0 technology enables a crucial change in authorship leading to *two-way communication* for better support and user-generated data. Finally, I will return to the concept of the empowered patient and question whether both patients and health professionals alike are ready for the change such a dynamic will present to their relationship.

To state that the Internet has brought about a revolution in information is hardly news. However, it is perhaps worth reminding ourselves just how massive a change this has been. Ten years ago, our postgraduate tutorials might have involved students critically analysing patient information leaflets for factors such as the most effective layout, typeface, and font size. Only a decade later our students, and many others with a stake in the health industry, are producing apps., podcasts (did such things exist in 2000?), and videos to provide patients with dynamic and vibrant information accessible through their “smart” phones and other hand-held devices (see, e.g., http://www.healthguru.com). Whether these new methods are any more effective in preparing patients for what they are about to experience has not been fully evaluated^1^; nevertheless, time and technology marches on and to present a patient with a sheet of text would no longer seem acceptable.

So the wide availability of digital technologies means information we provide to patients is now exciting and has greater accessibility. However, there is a more fundamental change in patient information brought about by the Internet and this concerns its direction of flow. Web 2.0 technology means that user-generated information has turned communication from monologue, that is, a unidirectional flow of information from health professional to patient, into dialogue. This dialogue may involve information flowing back from patient to health professional or may manifest as communication between patients themselves. Consequently a plethora of interactive patient websites has evolved. This appears to be particularly true in the USA where medical insurance companies and private health-care providers have flooded the market with forums, communities, and social networks to connect all those who would talk about health (see for instance http://www.patientslikeme.com). It could be argued that the fiscal model may be driving the development of such communities in the USA, as it benefits those who run these sites for people to talk about health; but whatever the motive, there is no doubt that patients value the support they get from being “digitally connected” to others in the same situation.^1^

The implication of Web 2.0 for imaging practitioners is twofold: First, it is now possible for our patients to communicate with us online, a facility made even more accessible thanks to the wide range of “instant” devices such as smart phones and tablets. Second, they can access one another. Therefore, providing a platform to support anxious patients through online dialogue may be easier than imagined and could prove far more effective in preparing them for their examination than traditional unidirectional approaches. This is because the patient is best placed to know what it is that is making them anxious and therefore what they want to know. For example, radiography academics at the University of Salford are working with clinical colleagues and talking to the National Health Service (Public Health England) to design a Digital Social Network for women attending for their first breast screening mammogram. The idea is simple. By talking to others who have had the experience, users will be better prepared for their first examination than by reading “static” information, whatever its format. There remains the question of how, and indeed whether, such networks should be managed, for instance, to address extreme views and avoid scare mongering, and the work at Salford intends to explore these issues with both practitioners and users alike.

So, clearly, the wealth of information patients now have access to means they are likely to be more knowledgeable about their health. Indeed, we are seeing the emergence of the so-called “smart-patient,” and combined with Web 2.0 technology, such patients are being recruited to share their knowledge not only with other patients but with health professionals, even adopting the roles of educator and research collaborator (see http://www.smartpatients.com). But does it follow that more knowledgeable patients will automatically be more involved in decisions regarding their treatment and/or diagnostic pathways? The Open Notes project would suggest so. In this study, 20,000 patients in Boston, rural Pennsylvania, and Seattle were given full access to their notes via an Internet portal over the period of 1 year.^2^ The results showed that 90% of patients were in favour of having been able to read their notes, citing improved adherence to medications, and better control and involvement in decisions about their (and their family's) health. Physicians involved in this study were more reticent, however, with up to 30% of them admitting to having to take more care in wording the notes because patients were viewing them.

Critically, therefore, the data from the Open Notes project also hints at a battle for power. A third of patients wanted to be able to “approve” notes, whereas around 90% of physicians disagreed with such a move.^3^ Therefore, technology and the Internet may be able to provide health professionals and patients with the means to move towards transparency for shared clinical decision making. However, managing the shifting power balance in the patient–practitioner relationship needs much more consideration. A study at Salford, which looked at health professionals as service users in the UK National Health Service Breast Screening Service, showed that it is not only health professionals who show resistance to such a change. In our study, “professionally-educated patients” said they would be reluctant to question mammographers regarding compression used during the examination because “the expert knows best.”^4^ We therefore suggest that some patients may choose to remain disempowered, finding the cultural shift required to bring about the patient–clinician relationship too difficult to make, and it is possible that such reticence may be more acutely felt in patients from hierarchical cultures and/or older generations where there is a strong tradition for respecting the “white coat.”

So where does all this leave us? Our patients will, without doubt, become more knowledgeable about what to expect, be this is as a result of what we provide or what they can quite easily access on the Internet (although the former is preferable if we are to help them negotiate the myriad poor quality information out there). However, as professionals are we prepared for how this might influence our relationship with patients? Are we ready for a patient who may challenge our choice of imaging modality, technique, or contrast medium because their knowledge of their own disease is superior to our own? And if we really are signed up to the notion of shared decision making, how do we help patients develop the confidence to make such decisions? Perhaps Francis Bacon got it wrong; knowledge is not power but only has the potential to be so. We also require the leadership to understand the value of, embrace and manage, such a change in the balance of power, and health-care curricula must promote the development of these attitudes and skills.
